# Modulation of the Senescence-Associated Inflammatory Phenotype in Human Fibroblasts by Olive Phenols

**DOI:** 10.3390/ijms18112275

**Published:** 2017-10-30

**Authors:** Beatrice Menicacci, Caterina Cipriani, Francesca Margheri, Alessandra Mocali, Lisa Giovannelli

**Affiliations:** 1 Department of Biomedical, Experimental and Clinical Science Mario Serio, Experimental Pathology and Oncology Section, University of Florence, Viale Morgagni 50, 50134 Florence, Italy; beatrice.menicacci@stud.unifi.it (B.M.); fmargheri@unifi.it (F.M.); 2 Department of Medical Biotechnologies, University of Siena, 53100 Siena, Italy; 3 NEUROFARBA Department, Pharmacology and Toxicology Section, University of Florence, Viale Pieraccini 6, 50139 Florence, Italy; caterinacipriani8@gmail.com (C.C.); lisa.giovannelli@unifi.it (L.G.)

**Keywords:** oleuropein, hydroxytyrosol, replicative senescence, SASP, inflammatory phenotype

## Abstract

Senescent cells display an increase in the secretion of growth factors, inflammatory cytokines and proteolytic enzymes, termed the “senescence-associated-secretory-phenotype” (SASP), playing a major role in many age-related diseases. The phenolic compounds present in extra-virgin olive oil are inhibitors of oxidative damage and have been reported to play a protective role in inflammation-related diseases. Particularly, hydroxytyrosol and oleuropein are the most abundant and more extensively studied. Pre-senescent human lung (MRC5) and neonatal human dermal (NHDF) fibroblasts were used as cellular model to evaluate the effect of chronic (4–6 weeks) treatment with 1 μM hydroxytyrosol (HT) or 10 μM oleuropein aglycone (OLE) on senescence/inflammation markers. Both phenols were effective in reducing β-galactosidase-positive cell number and p16 protein expression. In addition, senescence/inflammation markers such as IL-6 and metalloprotease secretion, and Ciclooxigenase type 2 (COX-2) and α-smooth-actin levels were reduced by phenol treatments. In NHDF, COX-2 expression, Nuclear Factor κ-light-chain-enhancer of activated B cells (NFκB) protein level and nuclear localization were augmented with culture senescence and decreased by OLE and HT treatment. Furthermore, the inflammatory effect of Tumor Necrosis Factor α (TNFα) exposure was almost completely abolished in OLE- and HT-pre-treated NHDF. Thus, the modulation of the senescence-associated inflammatory phenotype might be an important mechanism underlying the beneficial effects of olive oil phenols.

## 1. Introduction

Cellular senescence is characterized by complex modifications in the protein expression profile of the cell, leading to replicative arrest and cell dysfunction [1]. In particular, senescent cells are characterized by a strong increase in the secretion of growth factors, inflammatory cytokines, and proteolytic enzymes, termed the “senescence-associated secretory phenotype” (SASP), that has been thoroughly described in cultured fibroblasts [2]. The SASP is thought to play a major role in many age-related diseases, such as Alzheimer’s disease [3], cancer [4], where it contributes to the maintenance of a chronic inflammation which is typical of this pathology [5], and endothelial dysfunction in cardiovascular diseases and type 2 diabetes, also characterized by a low-grade chronic inflammation state [6]. Thus, interest has been growing on the possibility of modulating the SASP with pharmacological or dietary interventions.

Olive oil is an important component of the Mediterranean diet, to which many positive health effects have been attributed. In the area of aging and age-related diseases, observational studies indicate that olive oil intake is associated with reduced cardiovascular risk [7] and mortality [8], and improved mental and cognitive state in aged subjects [9]. Olive oil intake has also been shown to reduce inflammation and Nuclear Factor kappa-light-chain-enhancer of activated B cells (NFκB) activation in peripheral blood leukocytes in healthy human subjects [10,11]. Studies in rodent models of normal [12] and accelerated aging [13] have shown improvement in age-related dysfunction upon administration of olive oil phenolic compounds, which have been proposed as candidates to counteract age-associated neurodegeneration [13]. Mechanistic studies indicate that these compounds are able to act at different sites, interfering with protein function and gene expression modulation to modify cellular pathways relevant to the aging process, including the inflammatory ones [14,15]. Drupes and leaves of Olea europaea contain high amounts of phenolic compounds reported to play a protective role in inflammation-related diseases. Among these, hydroxytyrosol and oleuropein are the most abundant and extensively studied. The anti-inflammatory activity of both these compounds has been documented in a number of in vitro and in vivo models, and repeatedly reviewed [16,17]. However, the anti-inflammatory activity of these compounds on the SASP has never been tested.

In the present work, we evaluated the effect of selected olive phenols on the senescence-associated secretory phenotype (SASP) in cultured pre-senescent human lung fibroblasts (MRC5), a well-known model of cellular senescence, and in neonatal human dermal fibroblasts (NHDF). Pre-senescent cultures were chronically treated with hydroxytyrosol (1 μM) or oleuropein aglycone (10 μM), and the development of both cellular senescence and SASP were evaluated at the end of the treatment period. These data indicate that the modulation of the senescence-associated inflammatory phenotype might be an important mechanism underlying the beneficial effects of olive phenols in aging.

## 2. Results

**Figure 1:**
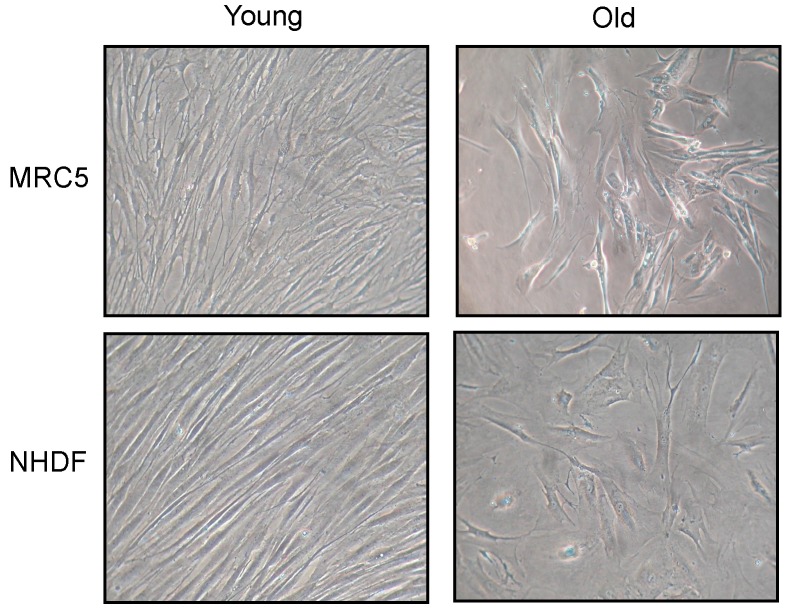
Fibroblast morphology after 1 week (**young**) and 2 weeks (**old**) in culture. Images are representative of cell cultures photographed using a phase-contrast microscope (100× magnification).

Changes in fibroblast morphology induced by cellular senescence are shown in Figure 1. Photos have been taken of both MRC5 and NHDF cell cultures, at low (young) and high (old) population doubling level (PDL). Old fibroblasts, in both cell types, showed a large flattened morphology, also evident for nuclear shape, compared to young. In addition, old cultures did not reach confluence after 15 days from seeding.

**Figure 2:**
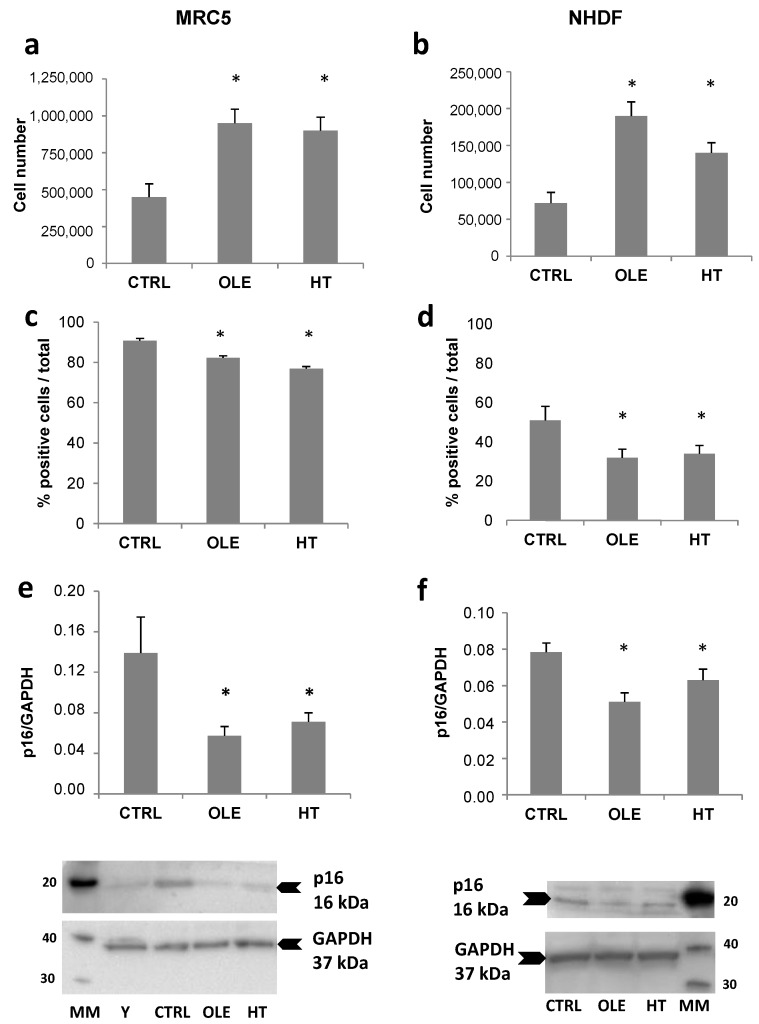
Effect of olive phenols on cellular senescence of pre-senescent human lung (MRC5) (left) and human dermal (NHD) (right) fibroblasts. Cells were treated for 4–6 weeks with 10 μM oleuropein (OLE) or 1 μM hydroxytyrosol (HT). (**a**,**b**) number of cells recovered at the end of the treatment; (**c**,**d**) percentage of beta-galactosidase-positive cells; (**e**,**f**) p16 protein level (ratio of the internal reference Glucose 6 Phosphate Dehydrogenase (GAPDH) enzyme) measured by western blot. Examples of p16-and GAPDH-blotted membranes are shown below the respective western blot graphs: MM = Magic Mark (molecular weight reference, with the corresponding kDa reported at the side of each band); Y = sample from young MRC5 cells showing very little expression). Arrows indicate the position of the specific band used for quantification. Bars are the mean ± standard error (SE) of 3 experiments. * *p* < 0.05 vs. senescent untreated cells (CTRL).

The effect of the long-term (4–6 weeks) treatment with olive phenols on senescence development is shown in Figure 2. The parameters assessed in order to evaluate senescence were: the total number of cells collected and counted at the end of the treatment, the percentage of cells positive to SA-βgal staining, and the level of p16 protein expression. Both hydroxytyrosol (HT) and oleuropein aglycone (OLE) brought about a significant increment in cell number, while SA-βgal staining and p16 protein levels were reduced. It can be noticed that MRC5 and NHDF cells exhibited a different degree of SA-βgal staining, in agreement with the different stages of the cultures, that is, fully senescent and non-cycling for MRC5 and still cycling and pre-senescent for NHDF. The reduction of SA-βgal staining appeared stronger in the proliferating NHDF compared to MRC5, whereas on the contrary p16 expression was reduced to a greater extent in MRC5 than in NHDF.

**Figure 3:**
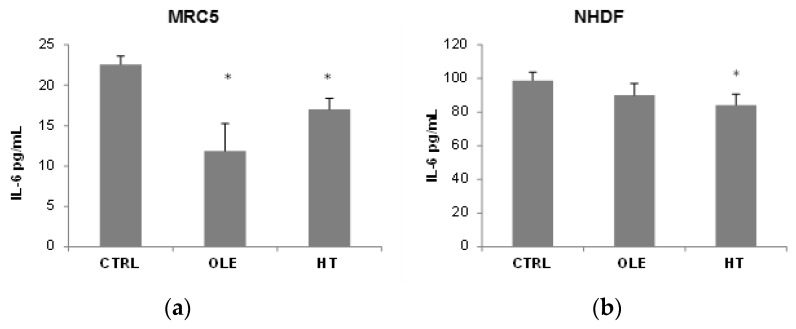
Effect of olive phenols on IL-6 release in the extracellular medium conditioned by MRC5 (**a**) and neonatal human dermal (NHDF) (**b**). Cells were treated for 4–6 weeks with 10 μM oleuropein (OLE) or 1 μM hydroxytyrosol (HT). IL-6 levels were measured in 24 h conditioned cell media by ELISA method. Bars are mean ± SE of 3 experiments. * *p* < 0.05 vs. senescent untreated cells (CTRL).

We then addressed the question whether OLE and HT were able to also reduce the SASP, and used IL-6 release in the extracellular medium as a SASP marker (Figure 3). In MRC5 fibroblasts, OLE reduced IL-6 release to about a half after 4–6 weeks of treatment, while the effect of HT was less strong although statistically significant. A shorter 2 weeks treatment did not have effects on IL-6 release (data not shown). In NHDF, a tendency towards a reduction was seen, however only HT treatment reached statistical significance. It is of notice that the basal IL-6 release (CTRL) was 5 time higher in NHDF than in MRC5.

**Figure 4:**
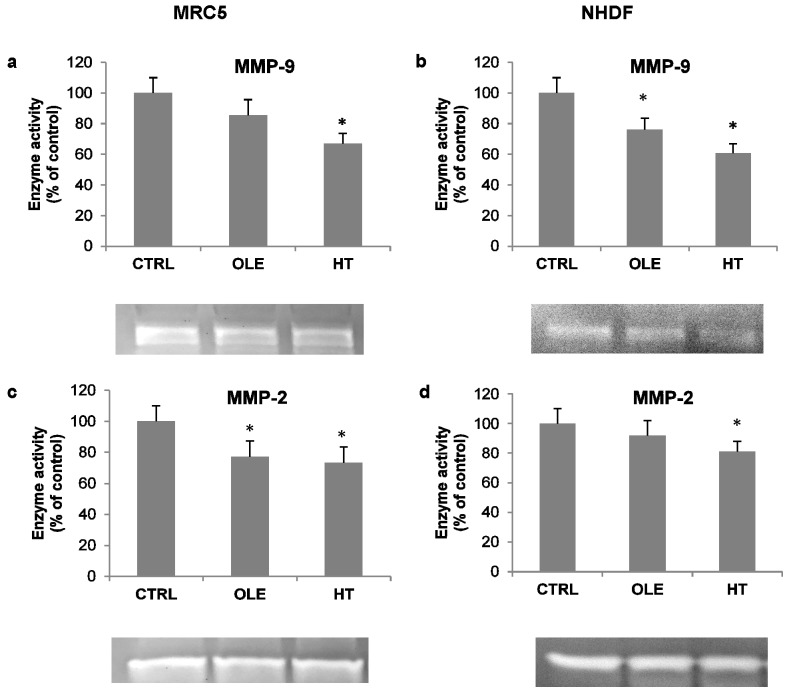
Effect of olive phenols on metallo-protease (MMP) activity in the extracellular conditioned medium from MRC5 (left) and NHDF (right) cells. Cells were treated for 4–6 weeks with 10 μM oleuropein (OLE) or 1 μM hydroxytyrosol (HT), and gelatinolytic activities were evaluated by gelatin zymography in 24 h conditioned cell media. (**a**,**b**) MMP-9; (**c**,**d**) MMP-2. Bars are mean ± SE of 3 experiments. Examples of zymography gel bands are shown below each graph. * *p* < 0.05 vs. senescent untreated cells (CTRL).

As an additional index of inflammatory activation, the activity of released metalloproteases was also assessed in cell-conditioned media, by means of gelatin zymography. Under these conditions, the MMP-9 and MMP-2 bands were evident (Figure 4, zymogram images). Quantification analyses of bands showed that OLE and HT induced a decrease in the activity of both metallo-proteases (MMPs): the effect was more prominent on MMP-9 with HT treatment (−30% in MRC5 and −40% in NHDF, compared to respective controls).

**Figure 5:**
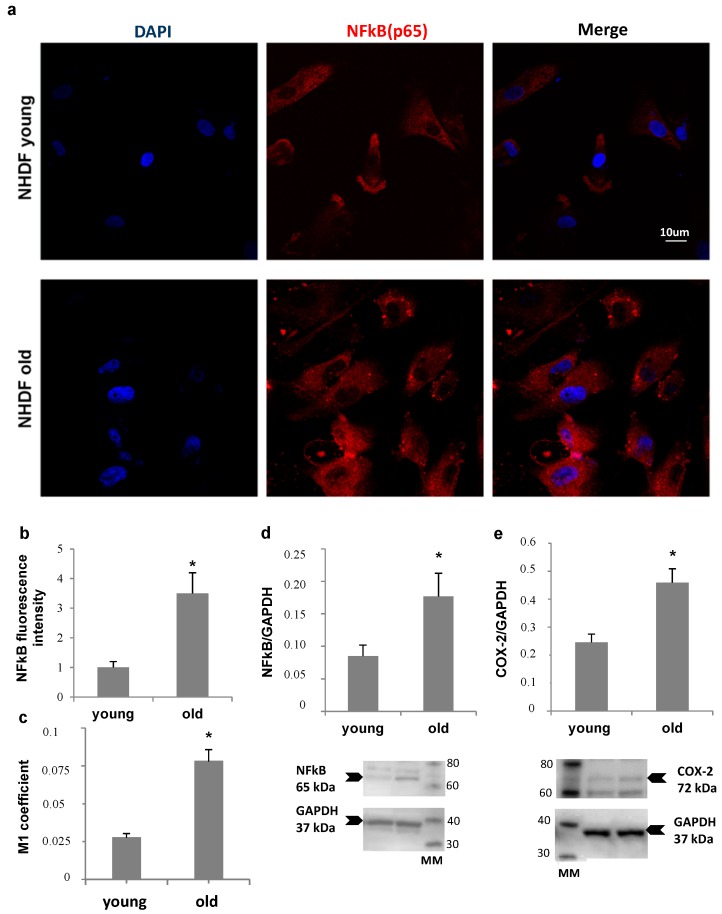
Nuclear Factor kappa-light-chain-enhancer of activated B cells (NFκB) and Ciclooxigenase type 2 (COX-2) proteins in young (Population Doubling Level (PDL) 10) and old (PDL 35) NHDF cultures. NFκB intracellular localization analyzed by immunofluorescence (**a**). Histograms show NFκB fluorescence quantification (**b**) and nuclear localization (NFκB/DAPI) by Mander’s coefficient (M1, (**c**)) using Image J software. NFκB p65 (**d**) and COX-2 protein expression (**e**) quantification through Western Blot analyses. Bars are the mean ± SE of 3 experiments. Examples of NFκB-, COX-2- and GAPDH-blotted membranes are shown below the respective western blot graphs: MM = Magic Mark (molecular weight reference, with the corresponding kDa reported at the side of each band). Arrows indicate the position of the specific band used for quantification. * *p* < 0.05 vs. young fibroblasts.

NHDF fibroblasts, at low and high PDL levels, were also compared for NFκB expression and nuclear translocation, and for Ciclooxigenase type 2 (COX-2) levels, as markers of inflammatory status (Figure 5). Panel a shows an increase in nuclear localization of NFκB in old fibroblasts compared to young, confirmed by Mander’s coefficient increment from about 0.025 to 0.075 (Panel c), while an about four-fold increase in total NFκB fluorescence was detected by quantitative image analysis (Panel b). Accordingly, a more than double increase in NFκB protein level (Panel d) was measured by Western Blot analysis in old cells compared to young. In addition, COX-2 protein level was two-fold increase in old fibroblast lysates (Panel e).

**Figure 6:**
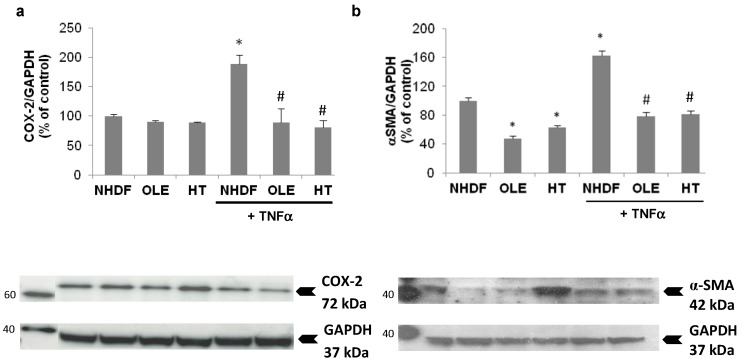
Effect of olive phenols on COX-2 (**a**) and α-Smooth Muscle Actin (α-SMA) expression (**b**) in senescent (PDL 35) NHDF, in basal and stimulated conditions. Cells treated for 4–6 weeks with 10 μM oleuropein (OLE) or 1 μM hydroxytyrosol (HT) were incubated for 3 h with 10 ng/mL of Tumor Necrosis Factor α (TNFα) or left untreated, then proteins were collected for Western Blot analyses. COX-2 (**a**) and α-SMA (**b**) protein levels (ratio of the internal reference GAPDH enzyme) are expressed as % of the level measured in unstimulated senescent cells (NHDF). Examples of immunodetected bands for COX-2 and α-SMA, together with GAPDH as loading control, are shown below the respective graphs. MM = Magic Mark (molecular weight reference, with the corresponding kDa reported at the side of each band). Arrows indicate the position of the specific band used for quantification. Bars are the mean ± SE of 3 experiments. * *p* < 0.05 vs. senescent untreated and unstimulated cells (NHDF). ^#^ *p* < 0.05 vs. senescent TNFα stimulated cells (NHDF + TNFα).

In a sub-group of experiments we aimed at evaluating the effect of the treatment with OLE and HT on externally-induced inflammation, using a 3 h Tumor Necrosis Factor α (TNFα) stimulation in high PDL (old) NHDF. The stimulation with exogenous TNFα brought about a further, two-fold increase of inducible COX expression, accompanied by a 60% increase in α-Smooth Muscle Actin (α-SMA) protein level (Figure 6). The long-term treatment with OLE or HT completely abolished the effect of TNFα on COX-2 and α-SMA expression; both were lowered to levels similar to those of control unstimulated cells.

**Figure 7:**
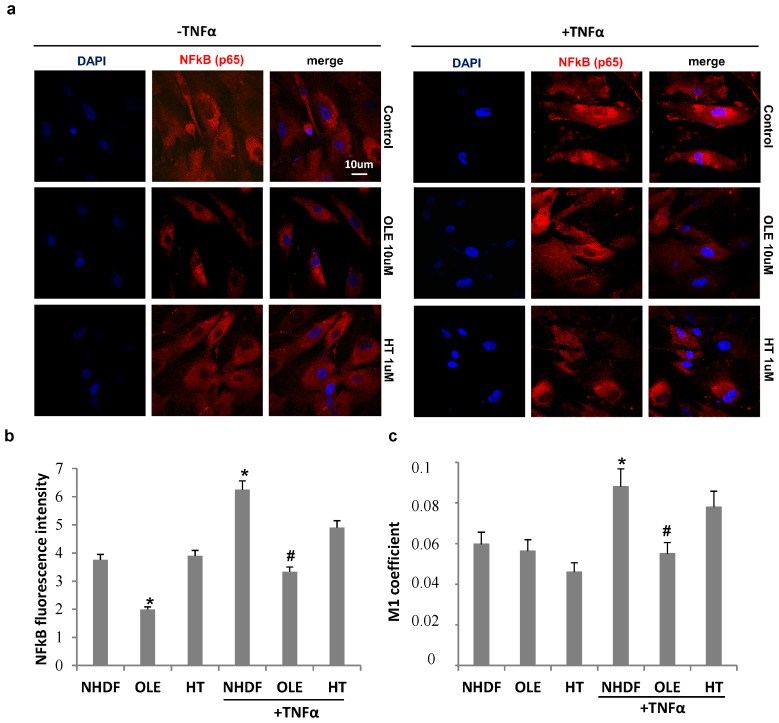
The effect of olive phenol pre-treatments on NFκB intracellular localization with and without TNFα stimulation. Old (PDL 35) NHDF cultures pre-treated with 10 μM oleuropein (OLE) or 1 μM hydroxytyrosol (HT), together with parallel control cultures, were incubated for 3 h with 10 ng/mL of TNFα or left untreated, then analized for NFkB localization by immunofluorescence (**a**). Total NFκB fluorescence intensity (**b**) and Mander’s coefficient (M1) (**c**), used to assess NFκB p65 colocalization with the nucleus (DAPI), were determined by means of ImageJ software. * *p* < 0.05 vs. senescent untreated and unstimulated cells (NHDF). ^#^ *p* < 0.05 vs. senescent TNFα stimulated cells (NHDF + TNFα).

The effect of olive phenol pre-treatments on NFκB intracellular localization with and without TNFα stimulation is reported in Figure 7. TNFα stimulation induced an increment in NFκB fluorescence intensity in old untreated cells and was lowered by both OLE and HT treatments, as showed by cell images (Figure 7a) and measured by image J software (Figure 7b). TNFα induced an about 30% increase in NFκB nuclear translocation, measured by Mander’s coefficient M1 (Figure 7c), in untreated cultures (NHDF), while both phenol pre-treatments counteracted the TNFα–induced nuclear transfer of NFκB, with OLE being more effective than HT. Accordingly, a change in cell morphology can also be observed after OLE pre-treatment, with a reduction in both nuclear and cell size (Figure 7a).

## 3. Discussion

The present results show that hydroxytyrosol and oleuropein, two phenols most abundant in olive fruit and derivatives, are able to attenuate the senescence-associated inflammatory phenotype, delaying at the same time cellular senescence in cultured human fibroblasts upon a long-term treatment. In fact, the anti-aging and anti-SASP activity was not detectable after 2 weeks, when cells did not yet show signs of senescence, indicating at the same time the ability of the tested compounds to counteract senescence-associated changes.

We studied two different human fibroblast strains. The first, fetal lung MRC5 fibroblasts, a consolidated model of cellular senescence, that were also used in previous studies of this research group, aimed to evaluate the anti-aging and anti-SASP effects of natural compounds [18,19]. The second, neonatal human dermal fibroblasts (NHDF), were studied as a useful model for the evaluation of dermal effects, and were characterized by slower development of senescence as compared to MRC5. Consequently, the two strains displayed a different degree of senescence at the end of the experiments: while MRC5 fibroblasts were fully senescent and replicatively blocked, NHDF still showed residual proliferative activity and a substantial percentage of SA-βgal-negative cells, although they also displayed the typical senescence-associated cellular and nuclear enlargement.

Chronic phenol treatments were effective in reducing the secretion in the extracellular medium of IL-6 and MMPs, markers of SASP and of a pro-inflammatory cell activity.

In view of the fact that NHDF cells showed a higher level of SASP compared to MRC5, as measured by means of IL-6 release into the extracellular medium [20], we decided to use the former as a cellular model to study in more detail the effect of the tested phenols on the inflammatory phenotype in senescent cells.

Progression towards senescence was accompanied by an increase in NFκB and COX-2 expression in these cells. Furthermore, NFκB was found to be increasingly translocated to the nucleus with senescence, in agreement with the pivotal role of this transcription factor pathway in driving the SASP [21]. It is accepted that senescent cells participate to the maintenance of a pro-inflammatory environment in ageing tissues, along with other stromal and immunity cells, and external stimuli [22,23]. When such an inflammatory micro-environment was mimicked by using TNFα stimulation of senescent cells, we observed a further increase in COX-2 induction, accompanied by increased expression of α-SMA, a marker of transition to myofibroblast phenotype. It has been shown that senescence and myofibroblast transition are connected, and notably, both senescent fibroblasts and myofibroblasts express α-SMA in cancer tissues, and a α-SMA-positive stroma is associated with worse prognosis in cancer [24]. NFκB nuclear translocation was also increased by TNFα exposure of senescent cells. However, in cells pretreated with OLE or HT, these inflammation-induced changes were almost completely abolished. Thus, olive phenols are able to modulate both the senescence- and the TNFα-induced inflammatory phenotype modulating early pivotal intracellular pathways such as NFκB signaling activation. It is increasingly recognized that NFκB activation is an initial stage in age-associated metabolic diseases, such as type 2 diabetes and atherosclerosis, and much research has focused on the possibility to pharmacologically target these processes in the search for new therapeutic approaches [25]. On the other hand, the chronic and slow-developing nature of these pathologies also leaves opportunities for prevention, and in this field olive-derived compounds might offer a most feasible possibility to slow the progressive development of low-grade chronic tissue inflammation by relatively simple dietary interventions.

It is very likely that an antioxidant action is involved in the effects we are describing. Inflammatory cytokine release may be induced by changes in intracellular redox status and recently it has been shown that olive phenols were able to reduce oxysterol-induced pro-inflammatory cytokine release in human peripheral blood mononuclear cells (PBMCs) along with reactive oxygen species (ROS) production and redox-based mitogen-activated protein kinase (MAPK) phosphorylation [26]. Furthermore, p16 expression has been linked to increased oxidative stress [27], and it is possible that the p16 level reduction we measured upon HT and OLE treatment is associated with lower ROS levels within the cells. We are planning to study this aspect more extensively in the future.

The micromolar concentrations used in the present in vitro work are in the lower range compared to most published studies [15]. However, data obtained in humans and experimental animals indicate that plasma concentrations of OLE and HT are in the nanomolar range in the hours following an acute administration, and that these compounds are extensively metabolized, mainly through conjugation [28,29]. So far, data on the biological effects of the metabolites are missing. Similarly, it is not known whether a prolonged exposure to these compounds, such as the one that we have used in our experiments, might lead to higher tissue levels. Aside from dietary consumption, it is feasible that appropriate formulations can substantially increase absorption and transfer to tissues, possibly approaching the micromolar range: this point will be matter of future development of this research work.

Food supplements based on olive leaf or fruit extracts have been long since popular, originally for the cardioprotective activity and more recently for an array of other beneficial effects. A common advantage of natural phenolic compounds deriving from fruit and vegetables is the fact that they are often current components of the human diet, devoid of important adverse effects. In the case of olive-derived biophenols, they have the additional advantage of being highly present in plant parts that are discarded during food preparation, such as leafs or olive mill solid and liquid waste, offering an opportunity for environment-friendly processing. The fact that these biophenols are able to counteract the senescence process, one of the hallmarks of aging [30] and to reduce the SASP, believed to be one important mediator of age-associated pathologies [31] further supports the preventive potential of these products for a healthier aging.

## 4. Materials and Methods

### 4.1. Olive Phenolic Compounds

Hydroxytyrosol (3,4-dihydroxy-phenylethanol) was obtained from Cayman Chemicals (Vinci Biochem, Vinci, Italy), and oleuropein from Extrasynthese (Lyon, France). To obtain the corresponding aglycone, oleuropein was subjected to a de-glycosilation procedure according to [32]. The aglycone obtained was referred to as OLE throughout the paper. Hydroxytyrosol was dissolved in ethanol (320 mM) and oleuropein in dimethyl sulfoxide (DMSO) (50 mM). Both the solutions were then serially diluted in culture media to obtain the final concentration, with the percentage of ethanol and DMSO in the medium being below 0.1%.

### 4.2. Cell Cultures and Treatments

MRC5 human fibroblasts from fetal lung tissue were purchased from NIA Aging Cell Repository (Coriell Institute, Camden, NJ, USA). Neonatal human dermal fibroblasts (NHDFs) were obtained from Lonza (Euroclone, Pero, Italy). All the cells were cultured in high-glucose (4500 g/L) Dulbecco’s Modified Eagle Medium (DMEM), supplemented with 10% fetal bovine serum, 2 mM l-glutamine, 100 units/mL penicillin, and 100 μg/mL streptomycin (Sigma-Aldrich, Milan, Italy) at 37 °C in 5% CO_2_ humidified atmosphere. At confluence, cultures were propagated by trypsinization, and the attained population doubling level (PDL) was calculated according to the equation: PDL = 3.32 × logN/N0 (where N and N0 are the recovered and seeded cell numbers, respectively).

The experiments were conducted starting from pre-senescent fibroblasts (PDL = 30 for MRC5 and 24 for NHDF) and terminated when the cells were either fully senescent (PDL = 40 for MRC5) or approaching senescence but still cycling (PDL 35 for NHDF). Senescence was assessed based on beta-galactosidase staining and on the inability of cells to complete one PDL after three consecutive weeks in culture. The cell cultures were treated continuously, from the beginning of the experiment to senescence, with 10 μM oleuropein (OLE) or 1 μM hydroxytyrosol (HT), and treatment duration ranged from 4 to 6 weeks. A shorter term treatment of 2 weeks was also evaluated in a single experiment with MRC5 cells. The culture medium was replaced every 2 days to maintain the concentration of the phenolic compounds in study relatively constant over time. Cells were propagated by splitting about 1:2, seeding into 25 cm^2^ flasks (250,000 cells per flask). Cell counts were started at PDL 35 for MRC5 and 31 for NHDF, recording the total number of obtained cells at each passage. At the end of the experiment, the cells were partly seeded in 6-well plates (100,000 cells/well), left until they reached sub-confluence (NHDF) or showed proliferative arrest (MRC5) and used for the experimental measurements: one set was used for protein extraction in RIPA buffer containing 1% protease and phosphatase inhibitor cocktail (Sigma-Aldrich Chemicals) with the aid of a cell scraper. The cell lysates were then sonicated, clarified by centrifugation and supernatants collected and stored at −20 °C. Protein content in the lysates was measured by using the Bio-Rad DC protein assay kit (Bio-Rad, Life Science, Segrate, Italy). A second set of parallel cultures was used for conditioned media (CM) collection: cell cultures were washed with fresh Phosphate-buffered saline (PBS), the medium replaced with DMEM containing 2% fetal bovine serum (FBS) and no treatment, and CM were collected after 24 h of incubation. These media were subsequently centrifuged at 250× *g* for 5 min and the supernatants aliquoted and stored at −20 °C for cytokine analysis and zymography. The medium volume/cell number proportion was 1 mL/1 × 10^5^ fibroblasts, normalized after counting cells recovered by trypsinization in parallel wells. Aliquots of control and treated cells were also seeded into 48-well dishes containing round coverslips (10,000 cells per well). These were partly fixed with 2% paraformaldehyde and 0.1% glutaraldehyde and used for SA-β-galactosidase staining, partly stimulated with TNFα (10 ng/mL) for 3 h in DMEM, fixed in 4% paraformaldehyde and then subjected to NFκB immunofluorescence as described below.

### 4.3. SA-β-Galactosidase Assay

SA-β-Gal staining was carried out by means of the Senescence b-Galactosidase Staining Kit (Cell Signaling Technology, Danvers, MA, USA). Staining was evident in 2–4 h and maximal in 12–16 h. The next day hematoxylin was used to counter-stain the cells. Cells were counted in a Bürker chamber to determine the percentage of SA-β-gal positive cells over the total.

### 4.4. Western Blot Analysis

Thirty-forty micrograms of lysate proteins for each sample together with the molecular weight Magic Mark (Invitrogen, Carlsbad, CA, USA) were subjected to 4–12% sodium dodecyl sulfate–polyacrylamide gel electrophoresis separation (Bis-Tris Plus BOLT, Invitrogen) and transferred to polyvinylidene fluoride membranes (PVDF, Millipore, Burlington, MA, USA). The membranes were blocked in 5% skim milk and incubated overnight with the following specific primary antibodies: rabbit anti-p16 (N-20, sc-467, Santa Cruz Biotechnology, Dallas, TX, USA), rabbit COX-2 (160126, Cayman Chemical, Ann Arbor, MI, USA), rabbit anti-NFkB (GTX102090, GeneTex, Irvine, CA, USA), mouse anti-α smooth actin (A2547, Sigma-Aldrich Chemicals, Italy), rabbit anti-Glucose 6 Phosphate Dehydrogenase (GAPDH) (14C10, Cell Signaling) followed by the suitable HRP-conjugated secondary antibodies (Sigma-Aldrich Chemicals). All resulting immunocomplexes were visualized with an enhanced chemiluminescence ECL detection system (GE Healthcare, Milano, Italy) and quantified by ImageJ software (NIH, Bethesda, MD, USA). Each density measure was normalized by using the corresponding GAPDH level as an internal control.

### 4.5. NF-kB Immunofluorescence

The primary antibody was the same than that used for western blotting, while the secondary was a Cy3–conjugated goat anti-rabbit IgG (Sigma–Aldrich Chemicals). The coverslips with the immune-labelled cells were mounted with an anti-fade mounting medium (Biomeda, Collegno, Italy) and analyzed under a Bio-Rad MRC 1024 ES confocal laser scanning microscope (Bio-Rad) equipped with a 15-mW Krypton/Argon laser source. The cells were observed with a Nikon Plan Apo X60 oil immersion objective (Nikon Instruments, Rome, Italy) at 595 nm. Series of optical sections (X- and Y-steps: 512 × 512 pixels) were then obtained through the depth of the cells, with a thickness of 1 μm at intervals of 0.8 μm (Z-step). A single composite image was obtained by superimposition of 20 optical sections for each sample. Total NFkB fluorescence intensity and Mander’s coefficient (M1), used to assess NFκB p65 colocalization with the nucleus (DAPI), were determined by ImageJ software.

### 4.6. IL-6 Detection in Conditioned Media

For the measurement of IL-6 content in cell-conditioned media, an ELISA kit (TMB Mini EDK 900-TM16, PeproTech, DBA, Milan, Italy), was used following the manufacturer’s instructions.

### 4.7. Gelatin Zymography of Conditioned Media

Conditioned media collected from MRC5 and NHDF fibroblasts (10–20 μL) were diluted with 4× Tris–Glycine SDS Native Sample Buffer (Invitrogen, Monza, Italy) and loaded onto 10% Novex Zymogram Gelatin Gels (Invitrogen). After electrophoretic separation, gels were developed following the manufacturer’s instructions. After incubation with SimplyBlue Safestain (Invitrogen) buffer, gelatinolytic activity was detected as transparent bands in the otherwise homogeneous blue gel and quantified using ImageJ software.

### 4.8. Statistical Analysis

The values are expressed as the mean ± standard error (SE). Statistical analyses of the data were performed using one-way ANOVA along with Bonferroni’s post hoc test. The Student’s *t* test was used for the analysis of paired data. *p* ≤ 0.05 was considered to be a statistically significant difference.

## 5. Conclusions

The chronic treatment with low doses of oleuropein and hydroxytyrosol is able to counteract the senescence process in human lung and dermal fibroblasts. The modulation of the senescence-associated inflammatory phenotype might be an important mechanism underlying the beneficial effects of olive oil phenols, suggesting their possible future employment as a dietary preventive approach to counteract age-associated pathologies.

## Abbreviations

NFκB	Nuclear Factor kappa-light-chain-enhancer of activated B cells
COX-2	Ciclooxigenase, type 2
TNFα	Tumor Necrosis Factor α
α-SMA	α-Smooth Muscle Actin
GAPDH	Glucose 6 Phosphate Dehydrogenase
